# Drug-Driven AMPA Receptor Redistribution Mimicked by Selective Dopamine Neuron Stimulation

**DOI:** 10.1371/journal.pone.0015870

**Published:** 2010-12-31

**Authors:** Matthew T. C. Brown, Camilla Bellone, Manuel Mameli, Gwenael Labouèbe, Christina Bocklisch, Bénédicte Balland, Lionel Dahan, Rafael Luján, Karl Deisseroth, Christian Lüscher

**Affiliations:** 1 Department of Basic Neurosciences, Medical Faculty, University of Geneva, Geneva, Switzerland; 2 Departamento de Ciencias Medicas, Facultad de Medicina, Universidad de Castilla-La Mancha, Albacete, Spain; 3 Departments of Bioengineering and Psychiatry and Behavioral Sciences, Stanford University, Stanford, California, United States of America; 4 Clinic of Neurology, Department of Clinical Neurosciences, Geneva University Hospital, Geneva, Switzerland; INSERM U901, France

## Abstract

**Background:**

Addictive drugs have in common that they cause surges in dopamine (DA) concentration in the mesolimbic reward system and elicit synaptic plasticity in DA neurons of the ventral tegmental area (VTA). Cocaine for example drives insertion of GluA2-lacking AMPA receptors (AMPARs) at glutamatergic synapes in DA neurons. However it remains elusive which molecular target of cocaine drives such AMPAR redistribution and whether other addictive drugs (morphine and nicotine) cause similar changes through their effects on the mesolimbic DA system.

**Methodology / Principal Findings:**

We used *in vitro* electrophysiological techniques in wild-type and transgenic mice to observe the modulation of excitatory inputs onto DA neurons by addictive drugs. To observe AMPAR redistribution, post-embedding immunohistochemistry for GluA2 AMPAR subunit was combined with electron microscopy. We also used a double-floxed AAV virus expressing channelrhodopsin together with a DAT Cre mouse line to selectively express ChR2 in VTA DA neurons. We find that in mice where the effect of cocaine on the dopamine transporter (DAT) is specifically blocked, AMPAR redistribution was absent following administration of the drug. Furthermore, addictive drugs known to increase dopamine levels cause a similar AMPAR redistribution. Finally, activating DA VTA neurons optogenetically is sufficient to drive insertion of GluA2-lacking AMPARs, mimicking the changes observed after a single injection of morphine, nicotine or cocaine.

**Conclusions / Significance:**

We propose the mesolimbic dopamine system as a point of convergence at which addictive drugs can alter neural circuits. We also show that direct activation of DA neurons is sufficient to drive AMPAR redistribution, which may be a mechanism associated with early steps of non-substance related addictions.

## Introduction

The VTA, which is the origin of the mesolimbic DA system, has been implicated in both the signaling of natural rewards and in the formation of drug addiction. Much previous data has shown that animals will readily self-administer electrical currents or addictive drugs into the VTA [1]. However due to the non-specificity of these interventions, it has been difficult to isolate the component that initiates the reinforcing behavior, which may eventually lead to addiction. Nevertheless, neurons of the VTA that release DA in target regions including the nucleus accumbens (NAc) and the prefrontal cortex as well as locally [2], [3] appear to be centrally involved.

Despite their diverse molecular targets, addictive drugs have in common that they increase mesolimbic DA levels [4]. One of the leading hypotheses posits that this surge in mesolimbic DA levels triggers synaptic adaptations, first in the VTA, which may be permissive for subsequent more general changes in other parts of the brain. Such circuit reorganization may eventually cause behavioral changes that underlie addiction.

According to the cellular mechanism engaged to increase DA levels, addictive drugs have been classified into three groups [5]. Opioids, cannabinoids, benzodiazepines [6] and the club drug gamma-hydroxybutyrate reduce transmitter release from inhibitory afferents onto DA neurons, indirectly increasing the firing rate of DA neurons, a mechanism defined as disinhibition. Nicotine, as a member of the second group, directly depolarizes DA neurons by activating alpha_4_beta _2_-containing acetylcholine receptors, whereas the third group, comprised of cocaine, ecstasy and amphetamines, targets the DAT. Despite the observation that the representatives of this third group decrease the firing frequency of the VTA neurons [7], [8] through D2 receptor mediated autoinhibition, extracellular DA levels actually surge [9]. This is due the block of the reuptake of the somato-dendritically released DA [10], [11].

Drug-evoked synaptic plasticity in the VTA appears at excitatory afferents onto DA neurons of the VTA already 24 h after a single injection of addictive drugs [12], [13]. In the case of cocaine it is induced by D1/D5 receptor [14] and NMDAR activation [12] and expressed in part by an insertion of GluA2-lacking AMPARs [15]. When rendered persistent through repetitive drug application, such adaptations in the VTA trigger synaptic plasticity downstream in the NAc [16], [17]. Several studies have identified the effects of cocaine on the DA system as a key contributor to its addictive properties. However, as cocaine also inhibits serotonin and noradrenaline uptake, it is unknown whether increased DA levels are crucial for cocaine-induced AMPAR redistribution. If that is the case, other addictive drugs should drive a similar receptor redistribution; even strong activation of VTA DA neurons alone may be sufficient.

Here we show that mice with a cocaine-insensitive DAT lack the redistribution of GluA2-lacking AMPARs following a single injection of cocaine. Furthermore, we demonstrate that single injections of addictive drugs with distinct mechanisms of action lead to a redistribution of AMPARs. Finally, we show that selective stimulation of DA VTA neurons at frequencies shown to increase DA levels in target regions [3] using channelrhodopsin mimics the AMPAR redistribution observed with addictive drugs.

## Results

### Cocaine-evoked AMPAR redistribution depends on DAT inhibition

**Figure 1:**
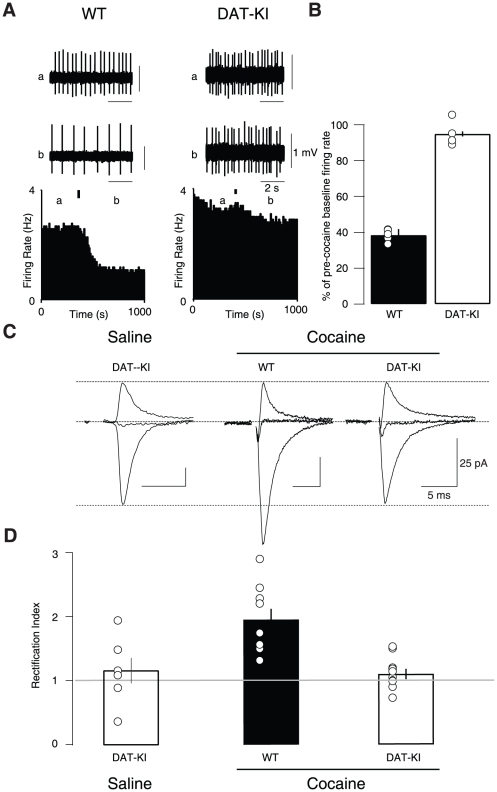
Cocaine drives the insertion of GluA2-lacking AMPARs via its effect on the DAT. (**A**) Single unit extracellular *in vivo* recordings (above) and corresponding firing rate plots (below) of VTA neurons during a single i.p. injection of 15 mg/kg cocaine in either WT (left) or DAT_KI_ (right) mice. Black bar denotes injection time, (a) and (b) denote points from which example traces were taken. (**B**) The resulting inhibition of neuron firing rate observed in WT mice (38±3.3%) was not present in DAT_KI_ mice (94.9±1.4%). n = 4–5, t_(7)_ = 16.5, p<0.0001. (**C**) Representative AMPAR excitatory postsynaptic currents recorded at −60, 0 and +30 mV (normalized to +40 mV AMPAR component) and RIs (**D**) of WT and DAT_KI_ mice 24 h post cocaine injection. Linearity corresponds to and RI of 1. Mean RI = 1.95±0.17 in WT, and 1.12±0.08 in DAT_KI_; F_(2–22)_ = 9.8, p<0.001, n = 5–9). All data are expressed as mean ± sem.

Cocaine is a non-selective monoamine reuptake inhibitor, and has been shown to bind the serotonin, noradrenaline and dopamine transporters with similar affinity [18]. To assess the importance of the action of cocaine on the DAT for AMPAR redistribution, we took advantage of a mouse line in which the DAT is still able to take up endogenous DA but rendered insensitive to cocaine (DAT_KI_ mouse, ref [19]. To validate this approach we injected cocaine intraperitoneally (i.p.) whilst recording from VTA neurons *in vivo* and observed an inhibition in the *in vivo* firing rate of VTA neurons in WT mice but not in the DAT_KI_ (Figure 1A, B). It is well established that the inhibition observed in the WT mice is due to the activation of D2 receptors present on the soma and dendrites of DA neurons. Nevertheless, DA levels remain elevated in the VTA and the NAc [7], [20] because the blockade of the DAT by cocaine predominates [10].

We then recorded AMPAR-mediated EPSCs and plotted the relative current–voltage (*I–V*)-relationship 24 h after a single injection of saline or cocaine in WT and DAT_KI_ mice. In the cocaine-treated DAT_KI_ mice, we found a linear *I–V* curve, similar to saline-injected DAT_KI_ mice (Figure 1C). To quantify the inward rectification, we calculated the rectification index (RI), which is the ratio of the slope of the *I–V* curve at positive divided by the slope at negative potentials. For GluA2-lacking AMPARs RI>1. This is due to the specific polyamine-sensitivity that inhibits the current flow at positive potentials [21]. As predicted from previous results, RI was significantly higher in WT controls injected with cocaine, reflecting the presence of GluA2-lacking AMPARs (Figure 1D). This finding confirms that the insertion of GluA2-lacking AMPARs is dependent on the inhibition of cocaine of the DAT.

### Addictive drugs trigger AMPA receptor redistribution

**Figure 2:**
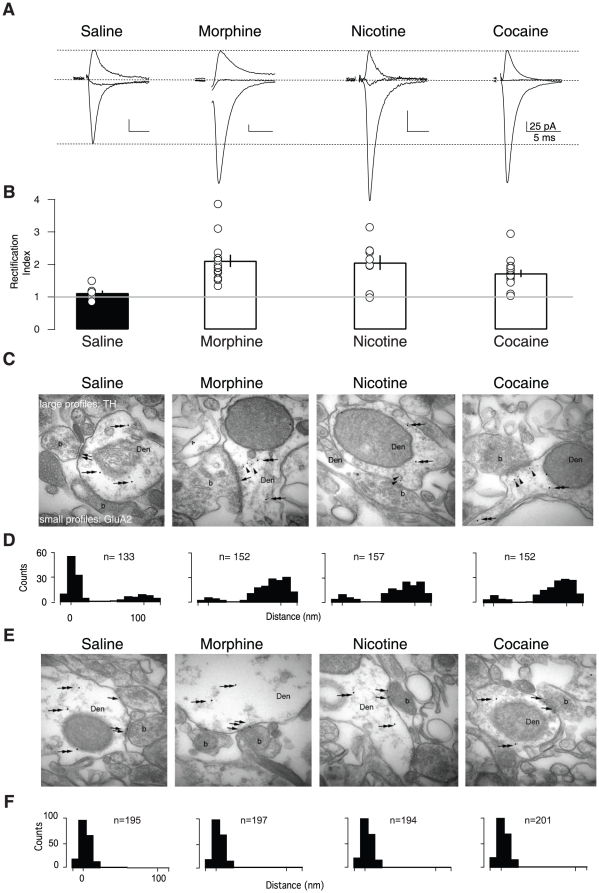
Addictive drugs cause rectification via AMPAR redistribution. (**A**) Representative traces of AMPAR excitatory postsynaptic currents recorded at −70, 0 and +40 mV. Examples are shown from recordings 24 h post injection. (**B**) Individual and averaged normalized rectification indices (RIs) (mean ± s.e.m) of saline and each drug treatment. RIs of morphine (2.12±0.27), nicotine (2.06±0.23) and cocaine (1.72±0.14) groups were significantly different from the saline (1.12±0.08) control group (F_(3,35)_ = 4.93, p<0.01, ANOVA. n = 7–15). (**C**) Representative electron micrographs of VTA sections from saline- or drug-treated animals. Large profiles (arrows) represent tyrosine hydroxylase (TH) immunoreactivity in dendrites (Den) forming asymmetrical synapses with boutons (b), and small profiles (arrowheads) represent GluA2 immunoreactivity. (**D**) Number of small profiles plotted against the distance from the postsynaptic density. (**E**) Same as in (C) but staining against PSD 95. (**F**) Same quantification as in (D) but for PSD 95.

Since these data suggest that the insertion of GluA2-lacking AMPARs is dependent on the cocaine-evoked surge in DA, other drugs known to increase DA levels could also drive this receptor redistribution. Indeed a previous study observed that these drugs can drive another form of synaptic change in the VTA [13]. We therefore tested whether a single exposure to morphine or nicotine, which increase DA in the mesolimbic system through distinct mechanisms [5], also drives the insertion of GluA2-lacking AMPARs. In such *ex vivo* slice recordings, we observed that the RI was significantly higher after the exposure to an addictive substance compared to saline-injected animals (Figure 2A, B). To demonstrate that the synaptic insertion of GluA2-lacking AMPARs occurred in exchange of native GluA2-containing receptors, we directly visualized the GluA2 subunit with post-embedding immunogold labeling at the electron microscopic level. In slices from saline-treated mice the majority of GluA2 labeling was observed at the synapse along with a small cytoplasmic pool. In slices from morphine, nicotine and cocaine-exposed mice the cytoplasmic GluA2 particles increased at the expense of the synaptic staining (Figure 2C, D). As a control, labeling of PSD95 was always observed at synaptic locations (Figure 2E, F). Thus we find that addictive drugs with diverse sites of action all can lead to AMPAR redistribution. A common feature of all these drugs is their ability to increase DA levels (either through increasing neuron activity or blocking DA reuptake). If this point of convergence is a critical component for the induction of AMPAR redistribution, then selectively stimulating DA VTA neurons in the absence of drugs should mimic this synaptic effect.

### Selective stimulation of VTA DA neurons mimics drug-driven AMPAR redistribution

**Figure 3:**
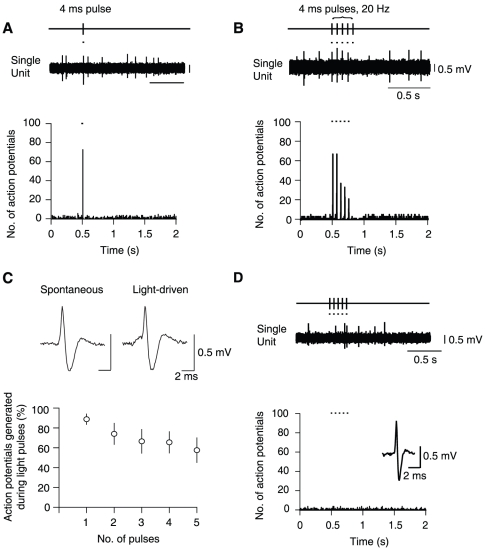
Light pulses are sufficient to mimic burst firing of dopamine (DA) neurons *in vivo*. (**A**) Representative single unit recording (above) and peristimulus time histogram (below, 5 ms bins) of a VTA DA neuron during a single light pulse (black markers; 4 ms, one sweep every 2 s) (n = 7). (**B**) The same VTA DA neuron as in (A) responding to 5 light pulses at 20 Hz. (**C**) Light pulses (black markers) are sufficient to drive action potentials, which do not differ in waveform characteristics from spontaneously occurring action potentials (above). Average percentage of action potentials generated by consecutive light pulses (below). Note the decrease in fidelity of action potential firing with increasing numbers of light pulses. (**D**) A GABAergic VTA neuron, which was recorded in close proximity to light-responsive DA neurons, exhibiting no response to five 4 ms light pulses (n = 7).

**Figure 4:**
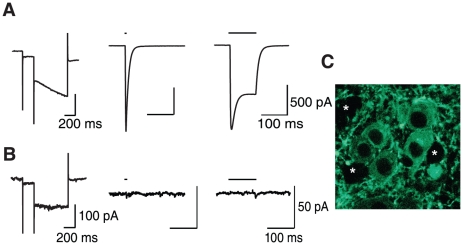
Expression of ChR2 causes light-activated currents in DA VTA neurons. (**A**) Representative whole cell voltage clamp recordings of a DA VTA neuron. Following identification of cell type by the presence of an *I*_h_ current (left) responses to light pulses (black lines) at 4 ms (middle) or 100 ms (right) in the presence of TTX were tested (n = 10). (**B**) Same as in (A) but a representative non-DA VTA neuron (note lack of *I*_h_ (left); n = 6). (**C**) Digital micrograph showing YFP labeling of neurons within the VTA, together with putative GABAergic unlabeled neurons (asterisks).

**Figure 5:**
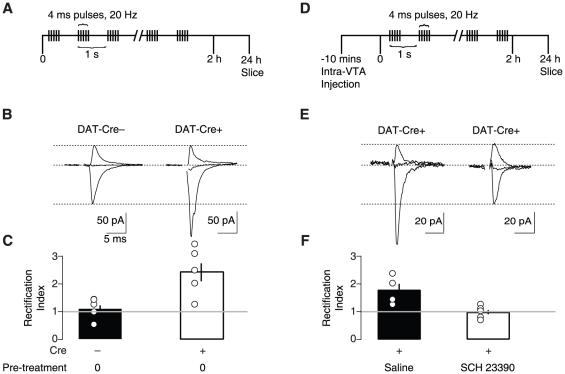
*In vivo* stimulation of dopamine neurons is sufficient to drive AMPAR redistribution. (**A**) Protocol of light stimulation *in vivo*. (**B**) Whole-cell voltage-clamp recordings made *ex vivo* 24 h post *in vivo* stimulation protocol. Representative traces of AMPAR excitatory postsynaptic currents recorded at −60, 0 and +30 mV (below). (**C**) Individual and averaged normalized rectification indeces (RIs) (mean ± s.e.m) following light stimulation. RI of DAT Cre− = 1.12±0.14 (n = 5). RI of DAT Cre+ = 2.45±0.32 (n = 5). p<0.01, Mann-Whitney *U* Test. (D) Protocol of intra-VTA infusion and light stimulation *in vivo*, (**E**) Same as in (B) following intra-VTA infusion and light stimulation, (**F**) Same as in (C). RI of DAT-Cre+ saline injected = 1.79±0.23 (n = 4). RI of DAT Cre+ SCH23390 injected = 0.99±0.09 (n = 5). p<0.05. Error bars represent s.e.m. Error bars are smaller than the symbol for some data points.

To assess the ability of VTA DA neuron activation to induce AMPAR redistribution, we virally expressed channelrhodopsin 2 (ChR2) selectively in DA neurons *in vivo* (stereotactic injection of AAV2 vectors with ChR2 flanked by double loxP sites into the VTA of DAT-Cre mice, see methods). We then lowered an optic fiber, connected to a blue light solid-state laser (473 nm), to drive action potentials in VTA DA neurons *in vivo* with brief pulses of light. We observed that VTA DA neurons expressing ChR2 fired action potentials immediately following five light pulses at 20 Hz (Figure 3A–C), whilst non-DA VTA neurons exhibited no light-evoked response (Figure 3D). This pattern of firing is similar to the burst firing of DA neurons recorded during rewarding stimuli or following administration of some addictive drugs [22], [23], [24]. *Ex vivo* slice recordings confirmed the presence of ChR2-induced photocurrents in DA neurons of DAT-Cre+ mice but not of non-DA neurons (Figure 4A–C). Injected animals were then exposed to an intermittent light stimulation protocol (Figure 5A. 5 pulses at 20 Hz each second) for 2 h. This duration mimics the time course of increased DA levels observed with a single dose of cocaine or nicotine [20], [25]. In whole-cell recordings *ex vivo* one day after the light simulation protocol, we obtained *I-V* curves from DA neurons and found that the RI was significantly higher in slices from DAT-Cre+ mice with respect to DAT-Cre− controls (Figure 5B, C). In a different set of experiments, we then applied the above stimulation protocol immediately following an infusion of SCH23390, a D1 receptor antagonist (Figure 5D). Under these conditions the RI was significantly lower that in control experiments where saline was infused (Figure 5E, F). Taken together, these experiments show that a strong stimulation of DA neurons, shown to efficiently release DA [3] and mimicking the time course of the drug action is sufficient to drive AMPAR redistribution at excitatory afferents onto DA neurons through a D1 receptor-dependent mechanism.

## Discussion

In the present study we show that the cocaine-driven redistribution of AMPARs depends on its effect on the DAT. We also show that morphine and nicotine, despite their distinct cellular effects on the VTA, cause a similar synaptic adaptation. Finally, in the absence of any pharmacological intervention, we use optogenetic tools to selectively drive the activity of DA neurons, and observe that this manipulation causes the AMPAR redistribution. We conclude that, as with addictive drugs, selective activation of the DA system is required to induce the insertion of GluA2-lacking AMPARs.

The observation that the cocaine-induced AMPAR redistribution is dependent on the action of cocaine on the DAT demonstrates that, despite the many other targets and actions of cocaine, the increased DA levels following cocaine administration are necessary for the AMPAR redistribution. Previous studies have suggested that, in a mouse line with a constitutive DAT knockout, cocaine self-administration can still be established [26], [27] while the constitutive D1 receptor knockout mouse line showed no such behavior [28]. These disparities may stem from significant adaptive changes in a mouse in which the DAT is absent during development. The DAT_KI_ mouse line, however, provides a model in which the reuptake of dopamine is closer to the wild-type condition (plus 64%), than in the DAT knockout [26], [29], whilst the action of cocaine on the DAT is severely impaired [30]. DAT_KI_ mice do not self-administer cocaine and fail to develop conditioned place preference [30], [31] , which confirms that block of DA reuptake mediates reinforcing properties of the drug. Moreover, in DAT_KI_ mice, compensatory adaptations seem not to affect D2-like receptors [32], which may explain why we did not observe any change in baseline synaptic transmission.

That other addictive drugs also cause increases in DA levels, albeit through distinct mechanisms, presents an intriguing convergence that may ultimately be responsible for their addictive properties. Our finding that these addictive drugs also induce AMPAR redistribution, backed up by data showing that another synaptic change is common to these drugs [13], further implicates a common mechanism. The current finding that a single injection of nicotine can cause rectification has also recently been confirmed by another group [33]. With the ChR2 we mimicked the activity of drugs that can activate the DA neurons directly (e.g. nicotine) or through disinhibition (e.g. morphine). While this shows that selective DA neuron activation is sufficient to mimic drug-induced AMPAR redistribution, other drug-specific mechanisms may contribute. Cocaine for example, while actually causing a decrease in DA neuron firing rate, is also able to induce AMPAR redistribution. One possibility for this result is that the increased dopamine concentration is responsible for the induction of this plasticity. Our data suggest that DA signaling within the VTA is driving AMPAR redistribution. First, other reports have used fast scan voltammetry to show that similar optogenetic stimulation protocols produce large DA transients in VTA target regions [34], [35], [36]. Second, a previous study has shown that a change in the AMPA/NMDA ratio, induced following administration of addictive drugs, was blocked by application of a D1-like receptor antagonist [37]. Our data with local VTA infusion of the same antagonist confirms this requirement for D1 signaling. This observation is also of interest in the context of recent evidence that some DA neurons co-release glutamate [35], [36].

Further experiments will have to establish the necessity of DA neurons activation by inhibiting the DA neurons while giving a drug or testing for occlusion if the effect of the stimulation after drug exposure.

Since previous pharmacological [12] and genetic [38] manipulations also demonstrated the need for NMDARs on DA neurons, intrinsic glutamatergic transmission may also be required and future studies will have to identify the locus and hierarchy of the convergence of DA- and NMDA-signaling. As drug-triggered AMPAR redistribution has also been induced in a VTA slice preparation, this implies a mechanism restricted to the circuitry within the VTA [37]. Indeed, bursting of DA neurons is also particularly efficient at driving DA release within the VTA [3], [2]. However, whether or not reciprocal connections between glutamatergic or GABAergic nuclei and DA VTA neurons were potentiated with this protocol cannot be ruled out. Indeed it is possible that adaptations in the NAc may have an indirect effect on the VTA via the strong back-projection of this nucleus to the midbrain [39].

A previous report has shown that stimulation of DA neurons, albeit with a different protocol, leads to behavioral conditioning, such as conditioned place preference (CPP, [34]. This provides evidence that increasing DA neuron activity could be sufficient to drive this behavioral response, and so represents a reinforcing stimulus [40], [41]. However the relationship between mesolimbic DA, synaptic plasticity and behavior is complex. Earlier reports suggest that CPP can be observed with morphine in mice that lack efficient DA synthesis [42]. Moreover the cellular changes were not investigated in these studies. In an inducible, conditional mouse line lacking NMDARs in DA neurons, the insertion of GluA2-lacking AMPARs and conditioned place preference were dissociated. Cocaine-evoked unbiased CPP was not affected (but see[43] for results with a biased protocol) in the NMDAR-mutant mice where AMPAR redistribution was absent. However these mice did show reduced reinstatement [38] and cue-induced cocaine seeking [16]. Our finding that selective stimulation of DA VTA neurons leads to AMPAR redistribution therefore provides strong evidence that increased DA neuron activity is capable of modifying the network at the synaptic level.

Given that addictive drugs are chemically very diverse and each has a distinct molecular target, it is surprising that they induce symptoms that are indistinguishable. Our study provides proof of principle for an early point of convergence in the function of the DA neurons of the VTA. The release of mesolimbic DA seems critical for the induction of a form of synaptic plasticity that predicts long-term adaptations in the neural reward circuits. The fact that we were able to elicit AMPAR redistribution with passive drug administration or passive light-activation indicates the permissive nature of these events for addiction.

We believe that by proposing a site of initiation for the final common pathway, future research may lead to an unifying model including non-substance dependent addictions to gain further mechanistic insight, and propose rational therapies.

## Materials and Methods

### Ethics Statement

All experiments were carried out in accordance with the Institutional Animal Care and Use Committee of the University of Geneva and with permission of the cantonal authorities (Permit No. 1007/3592/2).

#### Subjects

Experimental procedures were conducted in C57/BL6, DAT_KI_ [19] and DAT-Cre [44] mice. Mice were house together except for those implanted with guide cannulae, which were housed separately.

### Intraperitoneal (i.p.) injections in mice

C57/BL6 or DAT_KI_ mice (P14–21, 8–11g bodyweight) were injected i.p. with cocaine (15 mg/kg), morphine (15 mg/kg), nicotine (0.5 mg/kg) or 0.9% saline with a 26G hypodermic needle to minimize stress.

### Stereotactic injection of ChR2-AAV

Injections of AAV-ChR2 [34] produced at the University of North Carolina (Vector Core Facility) were made in 7–9 g DAT-Cre mice for *ex vivo* experiments, and 15–20g mice for *in vivo* electrophysiological recordings and *in vivo* light stimulation. Anesthesia was induced and maintained with isoflurane (Baxter AG, Veinna, Austria). The animal was placed in a stereotaxic frame (Angle One; Leica, Germany) and craniotomies were performed over the VTA either unilaterally (for *in vivo* recordings and *in vivo* light stimulation) or bilaterally (for *ex vivo* experiments) using stereotaxic coordinates (AP −3.3, ML ±1.3, DV 4.4, 10° angle). Injections of AAV-ChR2 were carried out using graduated pipettes (Drummond Scientific Company, Broomall, PA), broken back to a tip diameter of 10–15 µm, at a rate of ∼1 µl/min. In all experiments the virus was allowed a minimum of 12 days to incubate before any other procedures were carried out.

### Cannula implantation

Following anesthesia and completion of the craniotomy (see above), three holes were drilled around the craniotomy into which screws were placed. After virus injections (see above), a guide cannula (Plastics One, Roanoke, VA) was lowered slowly into position using the same coordinates as for virus injection, and cemented in place using dental cement (Lang Dental MFG Company, Wheeling, IL) to encase the base of the guide cannula and the screws. Once the cement had dried, a dummy cannula (Plastics One) was placed inside the guide cannula to prevent infection.

### Slice electrophysiology

Horizontal 200–250 µm slices of mouse midbrain were prepared in cooled artificial cerebrospinal fluid (ACSF) containing (in mM) NaCl 119, KCl 2.5, MgCl 1.3, CaCl_2_ 2.5, Na_2_HPO_4_ 1.0, NaHCO_3_ 26.2 and glucose 11, bubbled with 95% O_2_ and 5% CO_2_. In older mice in which cannulation and *in vivo* light stimulation had been performed, slices were prepared in ACSF as above with the addition of (in mM) kynurenic acid 3, NaHCO_3_, sucrose 225, glucose 1.25 and MgCl_2_ 4.9. Slices were kept at 34°C for 1 h before being transferred to the recording chamber superfused with 2.5 ml min^−1^ ACSF. Visualized whole-cell voltage-clamp recording techniques were used to measure holding currents from neurons of the VTA, identified as the region medial to the medial terminal nucleus of the accessory optic tract. Where possible, DA neurons were identified by presence of a large *I*_h_ immediately after entering whole-cell configuration. For experiments in which AMPAR EPSCs and photocurrents were recorded, the internal solution contained (in mM) CsCl 130, NaCl 4, MgCl_2_ 2, EGTA 1.1, HEPES 5, Na_2_ATP 2, sodium creatine-phosphate 5, Na_3_GTP 0.6 and spermine 0.1. For experiments in which only photocurrents were recorded, the internal solution contained (in mM) potassium gluconate 130, MgCl_2_ 4, creatine phosphate 10, Na_2_ ATP 3.4, Na_3_ GTP 0.1, EGTA 1.1, HEPES 5. Traces were amplified, filtered at 5 kHz and digitized at 20 kHz. The liquid junction potential was small and so traces were not corrected. All experiments measuring the *I–V* relationship were carried out in the presence of picrotoxin (100 µM) and D,L-APV (100 µM) in order to block GABA_A_ and NMDA receptors, respectively. The holding potentials were −60/−70 mV, 0 mV and +30/+40 mV, and the access resistance was monitored by a hyperpolarizing step of −10 mV with each sweep, every 10 s. Experiments were discarded if the access resistance varied by more than 20%. Synaptic currents were evoked by stimuli (0.04–0.1 ms) at 0.1 Hz through bipolar stainless steel electrodes placed rostral to the VTA. The rectification was calculated by dividing the gradient of the slope at negative potentials by the gradient of the slope at positive potentials.

### Simultaneous *in vivo* electrophysiological recordings and light stimulation

Anesthesia was induced with choral hydrate (4% w/v, 480 mg/kg i.p.) and maintained with supplemental doses as required (4% w/v, 120 mg/kg i.p.). All wound margins were infiltrated with lidocaine (4%), and corneal dehydration was prevented with Viscotears (Novartis; Basel, Switzerland). Mice were placed into a stereotaxic frame (Angle One) and their temperature was maintained using a homeothermic heating blanket (Harvard Apparatus, Holliston, MA). Anesthesia was continually monitored by testing reflexes to gentle corneal stimulation and a cutaneous pinch.

A craniotomy was performed to expose the cortex overlying the left VTA, of a size large enough to accommodate the guide cannula and the recording electrode. The cannula was sterotaxically positioned and slowly lowered vertically into position at the dorsal boundary of the VTA. Dental cement (Lang Dental) was used to secure the cannula whilst taking care not to allow cement to cover the craniotomy. A fiber optic cable (Thorlabs, Munich, Germany) was then lowered through the guide cannula so that ∼50 µm of fiber protruded from the guide cannula, and attached to a 473 nm solid-state laser (Crystalaser, Reno, NV). Extracellular recordings of action potentials were made using a glass recording electrode with a tip diameter of ∼1–2 µm, filled with a saline solution (0.5M NaCl) containing Chicago Sky Blue dye (2% w/v) and lowered into the VTA at a 10° angle. A reference electrode was placed in the subcutaneous tissue. Electrical signals were AC-coupled, amplified (Neurodata IR 183; Neurodata Instruments Corp., New York, NY), and monitored in real time using an audiomonitor (custom made). Any 50 Hz noise was eliminated using a Humbug (Quest Scientific, Vancouver, Canada). Signals were digitized at 20 kHz (for waveform analysis) or 5 kHz and stored on hard disk using a custom-made program within IGOR (WaveMetrics, Lake Oswego, OR). The bandpass filter was set between 0.3 and 5 kHz. Action potentials always exhibited an initial positive deflection. The laser was controlled using the Igor program via a TTL box. Following electrophysiological recordings, Chicago Sky blue dye was iontophoretically injected into the recording site (Stoelting, Wood Dale, IL) for confirmation that the recordings were made within the VTA. Dopaminergic VTA neurons were identified based on their established electrophysiological properties. These criteria included a biphasic action potential ≥1.1 ms [45], and slow regular-irregular firing [46], [47].

### *In vivo* stimulation of VTA DA neurons

Virus-injected and cannulated animals were allowed a minimum of 12 days to recover, and for the virus to express in VTA DA neurons. The same solid-state laser used for *in vivo* electrophysiological recordings was used to carry out the *in vivo* stimulation protocol in awake mice. In a subset of experiments, an infusion of 0.25µg of SCH23390 into the VTA through the guide cannula in 0.9% µl NaCl just prior to light stimulation. A fiber optic cable (Thorlabs) was customized to enable the mouse to move freely during stimulation. Briefly, the plastic cap of a dummy cannula (Plastics One) was hollowed out and a hole of sufficient diameter for the fiber optic to pass through made in the top. This was threaded onto the fiber optic, one end of which was stripped to leave a 120 µm external diameter. The plastic portion of an internal cannula (Plastics One) was glued onto the fiber optic leaving enough fiber to reach the bottom of the guide cannula. The fiber was then lowered into the guide cannula on the mouse, and the hollowed-out dummy cannula cap screwed onto the guide cannula. This allowed the fiber optic to turn freely throughout the stimulation protocol, whilst ensuring a constant length of the fiber remained within the guide cannula. The fiber was connected to the laser, which delivered five 4 ms pulses per second at 20 Hz for 2 hours (controlled using a Master 8 (A.M.P.I.)). All stimulations were carried out in a controlled environment.

### Post-embedding immunohistochemistry

Slices of the VTA from saline- and drug-treated mice were cut at 500 µm and immersed in 4% paraformaldehyde, 0.1% glutaraldehyde and ∼0.2% picric acid made up in 0.1 M phosphate buffer (PB; pH 7.4) for 6 hours. Slices were incubated in 1 M sucrose/PBS solution overnight, slammed onto copper blocks cooled in liquid nitrogen and processed for osmium-free embedding. Briefly, slices were incubated for 40 minutes in 1% uranyl acetate, dehydrated in methanol and embedded in Unicryl resin (Electron Microscopic Sciences, PA, USA). Ultrathin sections (70–90 nm) from Unicryl-embedded blocks were incubated for 45 min on pioloform-coated nickel grids with drops of blocking solution consisting of 2% albumin in 0.05 M TBS, 0.9% NaCl, and 0.03% Triton X-100. The grids were transferred to solutions of GluA2 and TH or PSD-95 and TH antibodies at a final protein concentration of 10 µg/ml diluted in blocking solution overnight at room temperature. After several washes in TBS, grids were incubated for 2 h in drops of goat anti-mouse IgG and goat anti-rabbit IgG conjugated to 10 nm colloidal gold particles and 20 nm-colloidal gold particles, respectively (BioCell International, Cardiff, UK), each diluted 1∶80 in a 0.05 M TBS solution containing 2% normal human serum and 0.5% polyethylene glycol. Grids were then washed in TBS for 30 min and counterstained for electron microscopy with saturated aqueous uranyl acetate and lead citrate. Ultrastructural analyses were performed in a Jeol-1010 electron microscope.

### Statistical analyses

Compiled data are expressed as mean ± s.e.m. The level of significance was set at p = 0.05 as determined by the non-parametric Mann-Whitney *U* Test or the one-way ANOVA with Dunnett post-test.
